# Supplementary material for: Genetic variation in the social environment affects behavioral phenotypes of oxytocin receptor mutants in zebrafish
Source: eLife. 2020 Sep 9;9:e56973. doi: 10.7554/eLife.56973 (PMC7481002; doi:10.7554/eLife.56973)
Supplement: Transparent reporting form [file elife-56973-transrepform.docx]

***eLife’s* transparent reporting form**

We encourage authors to provide detailed information *within their submission* to facilitate the interpretation and replication of experiments. If you have any questions, please contact us: [editorial@elifesciences.org](mailto:editorial@elifesciences.org).

**Sample-size estimation**

- You should state whether an appropriate sample size was computed when the study was being designed
- You should state the statistical method of sample size computation and any required assumptions
- If no explicit power analysis was used, you should describe how you decided what sample (replicate) size (number) to use

Please outline where this information can be found within the submission (e.g., page numbers or figure legends), or explain why this information doesn’t apply to your submission:

Sample size was determined according to the accepted practice for behavioral assays, but no statistical methods were used to predetermine sample size. We used 9 heterogeneous groups (i.e. focal individual with different genotype from the remaining individuals in the shoal; mutant focal in WT shoals and WT focal in mutant shoals) and 15 homogeneous groups (i.e. focal individual with the same genotype of the remaining individuals in the shoal; mutant focal in mutant shoals and WT focal in WT shoals). The smaller sample size of heterogeneous groups is due to the need of genotyping all individuals in these groups to single out the focal individual.

**Replicates**

- You should report how often each experiment was performed
- You should include a definition of biological versus technical replication
- The data obtained should be provided and sufficient information should be provided to indicate the number of independent biological and/or technical replicates
- If you encountered any outliers, you should describe how these were handled
- Criteria for exclusion/inclusion of data should be clearly stated
- High-throughput sequence data should be uploaded before submission, with a private link for reviewers provided (these are available from both GEO and ArrayExpress)

Please outline where this information can be found within the submission (e.g., page numbers or figure legends), or explain why this information doesn’t apply to your submission:

We used groups (shoals) as a replicate unit and only one individual was tested per group. Each individual was only tested once, hence we have used biological replicates only. All behavioral measurements were automatically extracted from a video tracking software (EthoVision, Noldus), or from videoframes using the open source FIJI image-processing package. The observer was blind to the treatment while extracting behavioral measurements from videos. We encountered no outliers (an outlier was considered as a data-point that is more than 2 standard deviations from the mean).

**Statistical reporting**

- Statistical analysis methods should be described and justified
- Raw data should be presented in figures whenever informative to do so (typically when N per group is less than 10)
- For each experiment, you should identify the statistical tests used, exact values of N, definitions of center, methods of multiple test correction, and dispersion and precision measures (e.g., mean, median, SD, SEM, confidence intervals; and, for the major substantive results, a measure of effect size (e.g., Pearson's r, Cohen's d)
- Report exact p-values wherever possible alongside the summary statistics and 95% confidence intervals. These should be reported for all key questions and not only when the p-value is less than 0.05.

Data is presented as mean ± standard error of the mean (SEM) and analyzed using Data were analyzed using SPSS 25.0. All data sets were tested for departures from normality with Shapiro-Wilks test. Two factor univariate ANOVA were used for comparing multiple groups. All data sets were corrected for multiple comparisons. Tukey’s Test comparisons were used as post-hocs. Graphs were prepared with GraphPad software.

Please outline where this information can be found within the submission (e.g., page numbers or figure legends), or explain why this information doesn’t apply to your submission:

(For large datasets, or papers with a very large number of statistical tests, you may upload a single table file with tests, Ns, etc., with reference to page numbers in the manuscript.)

**Additional data files (“source data”)**

- We encourage you to upload relevant additional data files, such as numerical data that are represented as a graph in a figure, or as a summary table
- Where provided, these should be in the most useful format, and they can be uploaded as “Source data” files linked to a main figure or table
- Include model definition files including the full list of parameters used
- Include code used for data analysis (e.g., R, MatLab)
- Avoid stating that data files are “available upon request”

Please indicate the figures or tables for which source data files have been provided:

The Source data files for Figure 1 and Table 1 have been made publicly available at Dryad data repository under the reference: Oliveira, Rui et al. (2020), Genetic variation in the social environment affects behavioral phenotypes of oxytocin receptor mutants, Dryad, Dataset, <https://doi.org/10.5061/dryad.xwdbrv1bq>
